# Direct Effect of Remifentanil and Glycine Contained in Ultiva® on Nociceptive Transmission in the Spinal Cord: *In Vivo* and Slice Patch Clamp Analyses

**DOI:** 10.1371/journal.pone.0147339

**Published:** 2016-01-15

**Authors:** Makoto Sumie, Hiroaki Shiokawa, Ken Yamaura, Yuji Karashima, Sumio Hoka, Megumu Yoshimura

**Affiliations:** 1 Department of Anesthesiology and Critical Care Medicine, Graduate School of Medical Sciences, Kyushu University, Fukuoka, Japan; 2 Department of Anesthesiology and Critical Care Medicine, Kyushu University Hospital, Fukuoka, Japan; 3 Department of Anesthesiology, Fukuoka University School of Medicine, Fukuoka, Japan; 4 Division of Health Sciences, Graduate School of Health Sciences, Kumamoto Health Science University, Kumamoto, Japan; Xuzhou Medical College, CHINA

## Abstract

**Background:**

Ultiva® is commonly administered intravenously for analgesia during general anaesthesia and its main constituent remifentanil is an ultra-short-acting μ-opioid receptor agonist. Ultiva® is not approved for epidural or intrathecal use in clinical practice. Previous studies have reported that Ultiva® provokes opioid-induced hyperalgesia by interacting with spinal dorsal horn neurons. Ultiva® contains glycine, an inhibitory neurotransmitter but also an *N*-methyl-D-aspartate receptor co-activator. The presence of glycine in the formulation of Ultiva® potentially complicates its effects. We examined how Ultiva® directly affects nociceptive transmission in the spinal cord.

**Methods:**

We made patch-clamp recordings from substantia gelatinosa (SG) neurons in the adult rat spinal dorsal horn *in vivo* and in spinal cord slices. We perfused Ultiva® onto the SG neurons and analysed its effects on the membrane potentials and synaptic responses activated by noxious mechanical stimuli.

**Results:**

Bath application of Ultiva® hyperpolarized membrane potentials under current-clamp conditions and produced an outward current under voltage-clamp conditions. A barrage of excitatory postsynaptic currents (EPSCs) evoked by the stimuli was suppressed by Ultiva®. Miniature EPSCs (mEPSCs) were depressed in frequency but not amplitude. Ultiva®-induced outward currents and suppression of mEPSCs were not inhibited by the μ-opioid receptor antagonist naloxone, but were inhibited by the glycine receptor antagonist strychnine. The Ultiva®-induced currents demonstrated a specific equilibrium potential similar to glycine.

**Conclusions:**

We found that intrathecal administration of Ultiva® to SG neurons hyperpolarized membrane potentials and depressed presynaptic glutamate release predominantly through the activation of glycine receptors. No Ultiva®-induced excitatory effects were observed in SG neurons. Our results suggest different analgesic mechanisms of Ultiva® between intrathecal and intravenous administrations.

## Introduction

Ultiva® is commonly administered intravenously during general anaesthesia. Its main constituent is remifentanil, a potent short-acting μ-opioid receptor agonist. Although behavioural studies in rats suggest that intrathecal administration of remifentanil induces profound analgesia [1, 2], Ultiva® is not approved for epidural or intrathecal use in clinical practice.

The clinical formulation of Ultiva® contains glycine as an acidic buffer. Glycine is a major inhibitory neurotransmitter in the central nervous system, and is also an important *N*-methyl-D-aspartate (NMDA) receptor co-activator with glutamate [3, 4]; the latter action is proposed as a potential mechanism for opioid-induced hyperalgesia (OIH) [5]. It has also been suggested that remifentanil itself may directly enhance NMDA receptor-mediated responses in the dorsal horn and promote hyperalgesia [6].

Although these findings suggest that intrathecal administration of Ultiva® may have contrary pro-nociceptive and anti-nociceptive actions, the balance between the actions of remifentanil and glycine in the spinal cord, and their consequent influence on nociception, is not understood. We examined the action of intrathecal administration of Ultiva® on substantia gelatinosa (SG) neurons using whole cell patch-clamp recordings *in vivo* and in *ex vivo* slice preparations of the adult rat spinal cord.

## Materials and Methods

All experimental procedures were approved by the Ethics Committee on Animal Experimentation at Kumamoto Health Science University (Permit Number: 12–026) and Kyushu University (Permit Number: A26-235-0), Japan, in accordance with the ARRIVE guidelines and the Guidelines of the Japanese Physiological Society.

### *In vivo* preparations

Methods used to establish *in vivo* preparations were similar to those described previously [7–12]. Briefly, male 5–8-week-old Sprague Dawley rats (Kyudo Co. Ltd., Fukuoka, Japan) were anaesthetized with intraperitoneal urethane (1.2–1.5 g/kg). If a withdrawal reflex appeared, a supplemental dose of urethane was given during surgery and the data collection period. Rectal temperature was maintained between 36°C and 37°C by a heating pad placed beneath the animal. Supplemental oxygen was administered through a nose cone. Laminectomy was performed at the Th12-L2 level and the animal was placed in stereotaxic apparatus (Model SR-5R, Narishige, Tokyo, Japan). After opening the dura, the L3 or L4 dorsal root was gently shifted laterally to expose Lissauer’s tract using a small glass retractor, so that a patch electrode could be advanced into the SG from the surface of the spinal cord. The pia-arachnoid membrane was removed using microforceps to make a window large enough to allow the patch electrode to enter the spinal cord. The surface of the spinal cord was irrigated with Krebs solution (117 mM NaCl, 3.6 mM KCl, 2.5 mM CaCl_2_, 1.2 mM MgCl_2_, 1.2 mM NaH_2_PO_4_, 25 mM NaHCO_3_ and 11 mM glucose) saturated with 95% O_2_ and 5% CO_2_ at a rate of 10–15 ml/min. At the end of experiments, the rats were terminally anaesthetized with an overdose of urethane and were killed by exsanguination.

### Patch-clamp recordings from *in vivo* preparations

Patch electrodes were pulled from thin-walled borosilicate glass capillaries (outside diameter 1.5 mm; World Precision Instruments, Sarasota, FL, USA) using a puller (p-97; Sutter Instrument, Novato, CA, USA). Patch electrodes were filled with a potassium gluconate-based internal solution (potassium gluconate, 136 mM; KCl, 5 mM; CaCl_2_, 0.5 mM; MgCl_2_, 2 mM; EGTA, 5 mM; HEPES, 5 mM; and ATP-Mg, 5 mM; pH 7.2). A patch electrode with a resistance of 8–12 MΩ was advanced at an angle of 30–45° into the SG through the window using a micromanipulator (Model MP-1, Narishige). A Giga-ohm seal was formed with neurons at a depth of 30–150 μm from the surface of the spinal cord. This distance was identified to be within the SG using transverse slices obtained from the spinal cord of 5–8-week-old rats at the same lumbar level. The location and morphological features of the recorded cells were confirmed further in some instances by intrasomatic injection of biocytin after obtaining synaptic responses. The membrane patch was ruptured by a brief period of more negative pressure. As a result, a whole cell configuration was established. A holding potential of −60 mV was used to record excitatory postsynaptic currents (EPSCs). Excitatory postsynaptic potentials (EPSPs) were also recorded at resting membrane potentials. For cutaneous stimulation, the receptive field of a neuron was first determined by applying non-noxious stimuli with a paintbrush across the skin of the hind limb. Noxious mechanical stimuli were applied to the receptive field of the hind limb for 3 s using toothed forceps. The toothed forceps was clamped during skin pinching to confirm noxious stimuli. Signals were collected using a patch-clamp amplifier (Axopatch 200B, Axon Instruments, Union City, CA, USA) and digitized with an A/D converter (Digidata 1322A, Axon Instruments). Data were stored on a personal computer using the pCLAMP data acquisition program (version 10.2, Axon Instruments) and analysed using a software package (Mini Analysis, version 6.0.3; Synaptosoft Inc., Decator, GA, USA).

### Spinal cord slice preparations

Methods for obtaining adult rat spinal cord slice preparations were similar to those described previously [13]. Briefly, male 5–8-week-old Sprague Dawley rats were anaesthetized with intraperitoneal urethane (1.2–1.5 g⁄kg), and lumbosacral laminectomy was performed. The spinal cord from L1 to S3 was removed and placed in pre-oxygenated Krebs solution at 1°C to 3°C. The rats were immediately given an overdose of intraperitoneal urethane and killed by exsanguination. The dura mater, ventral roots, dorsal roots and the pia-arachnoid membrane of the spinal cord preparation were removed. The spinal cord was mounted on a microslicer (PRO 7; Dosaka Co. Ltd., Kyoto, Japan), and a 500-μm thick transverse slice was cut at L3 or L4. The slice was placed in the recording chamber and perfused with Krebs solution saturated with 95% O_2_ and 5% CO_2_ at a rate of 15–20 ml/min and maintained at 36°C ± 1°C.

### Patch-clamp recordings from spinal cord slice preparations

Whole-cell patch-clamp recordings were made from SG neurons as described in the *in vivo* section. The SG was visually identified as a distinct translucent band across the superficial dorsal horn under a dissecting microscope with transmitted illumination as reported previously [13]. Miniature excitatory postsynaptic currents (mEPSCs) were recorded in the presence of 1 μM tetrodotoxin (TTX). Triangle voltage ramp commands (ramp from -120 mV to -50 mV and back to -120 mV of 0.9 s duration each) from the holding potential of −70 mV or −50 mV were applied every 20 s during the recording to examine the current-voltage relationships of drug-induced currents. Current responses to voltage ramps before application of drug were subtracted from those during application. The resulting current ramps were plotted as a function of membrane potential and further analysed.

### Drug application

Drugs were dissolved in Krebs solution and applied by perfusion via a three-way stopcock without any change in either the perfusion rate or the temperature. The drugs used in this study were Ultiva®, 2 mg (Janssen Pharmaceutical K.K., Tokyo, Japan), TTX (Wako, Osaka, Japan), glycine (Wako) and naloxone hydrochloride (Wako). All drugs were diluted to their final concentration in Krebs solution immediately before use. A 2-mg Ultiva® vial contains 15 mg glycine as an adjunct. Ultiva® containing 50 μM remifentanil hydrochloride with 2 mM glycine was superfused on to spinal cord slice preparations *in vivo* and *ex vivo*. The plasma concentration of remifentanil achieved by an intravenous infusion of Ultiva® at 0.05–0.20 μg·kg^−1^ min^−1^ is approximately 1–5 ng/ml [14, 15]. Remifentanil has a rapid blood-brain equilibration half-time because of its rapid onset of action. Although the transit of remifentanil to the cerebrospinal fluid is not completely understood, the concentration of Ultiva® superfused was not determined from the plasma concentration, but from the effect obtained in a previous study of Ultiva® or pure remifentanil [5, 16].

### Statistical analysis

All numerical data are expressed as the mean ± standard error of the mean (SEM). Statistical significance was determined as P <0.05 using Student’s paired *t* tests. Dose-respons relationship was calculated by linear regression analysis uging GraphPad Prism 6 (GraphPad software Inc, San Diego, CA, USA). Cumulative probability plots were constructed for mEPSC amplitude and frequency, and were compared using the Kolmogorov-Smirnov test, which was undertaken with Mini Analysis 6.0.3 (Synaptsoft). In the electrophysiological data, n refers to the number of neurons studied. The liquid junction potential between Krebs and patch-pipette solutions was not corrected.

## Results

Stable whole-cell patch clamp recordings were obtained from 20 SG neurons identified *in vivo*, and from 48 SG neurons in spinal cord slice preparations. Neurons with resting membrane potentials more depolarized than -50 mV were discarded. In current-clamp mode, all neurons in *in vivo* preparations showed spontaneous EPSPs of 2.9 ± 0.4 mV in amplitude and 8.4 ± 0.9 Hz in frequency. In voltage-clamp mode, all neurons in *in vivo* preparations showed spontaneous EPSCs of 15.8 ± 3.2 pA in amplitude and 10.6 ± 2.1 Hz in frequency. Further, all neurons in spinal cord slice preparations showed mEPSCs of 8.3 ± 0.8 pA in amplitude and 5.4 ± 0.9 Hz in frequency in the presence of TTX (1 μM).

### Effects of Ultiva® on the membrane potentials or currents and synaptic responses in SG neurons elicited by noxious stimuli *in vivo*

**Fig 1:**
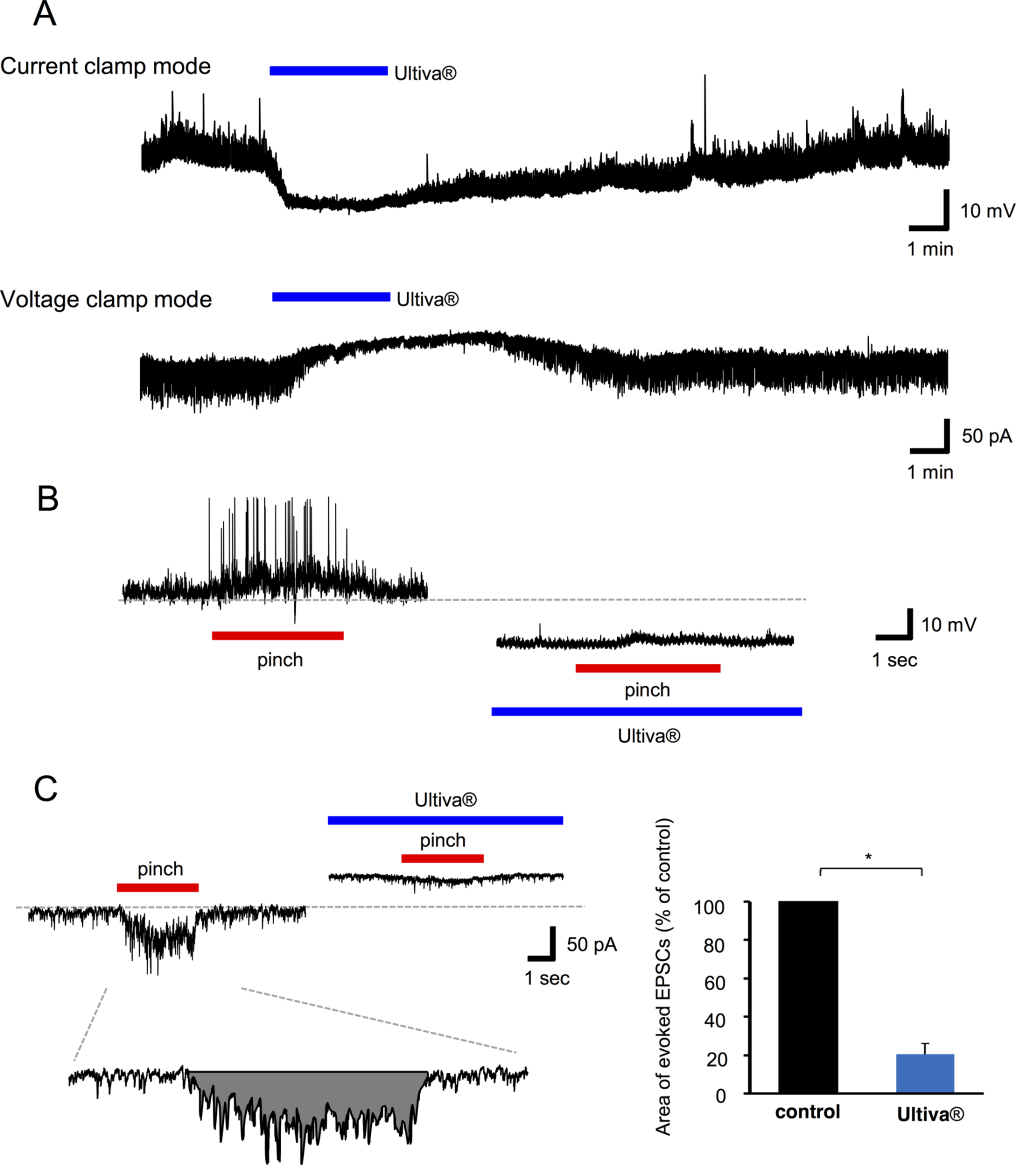
Effects of Ultiva® on pinch-evoked responses in substantia gelatinosa (SG) neurons *in vivo*. (A) Ultiva® (remifentanil 50 μM with glycine 2 mM) hyperpolarized SG neurons in current-clamp mode (top). Ultiva® induced an outward current in voltage-clamp mode (V_H_ = −60 mV, bottom). (B) Pinch stimuli applied to the ipsilateral hind limb produced a barrage of excitatory postsynaptic potentials (EPSPs) in current-clamp mode. Some of the EPSPs were large enough to initiate action potentials. The barrage of EPSPs was suppressed by Ultiva®. (C) Pinch stimuli applied to the ipsilateral hind limb produced a barrage of excitatory postsynaptic currents (EPSCs) in voltage-clamp mode. The barrage of EPSCs was suppressed by Ultiva® (upper left). Schematic diagrams of area surrounded by the baseline and border of EPSCs. Area was analysed by using Mini Analysis (lower left). Ultiva® suppressed the area of evoked EPSCs (n = 7, right). In this and subsequent figures, vertical lines accompanied by bars indicate the standard error of the mean and a statistically significant difference between groups (P <0.001) is indicated by an asterisk (*)

In current-clamp mode at membrane potentials of −60 mV to −65 mV, perfusion of Ultiva® on to the surface of the spinal cord hyperpolarized the cell membrane (−8.2 ± 1.1 mV, n = 11) in all neurons tested. In voltage-clamp mode at a holding potential of −60 mV, perfusion with Ultiva® produced an outward current in eight of nine neurons (27.7 ± 4.2 pA, n = 8; Fig 1A). In current-clamp mode, pinch stimuli applied to the receptive field of the ipsilateral hind limb elicited a barrage of EPSPs, some of which were large enough to elicit an action potential; the responses persisted during the stimulation. Perfusion with Ultiva® suppressed the pinch-evoked barrage of EPSPs and spontaneous EPSPs in all neurons tested (Fig 1B). Pinch stimuli also elicited a barrage of EPSCs in voltage-clamp mode. Perfusion with Ultiva® suppressed the evoked and spontaneous EPSCs in all neurons tested (Fig 1C, left). In the presence of Ultiva®, the area of evoked EPSCs was 20.4 ± 5.7% of the control area (n = 7, P <0.001; Fig 1C, right). These findings suggest that Ultiva® exerts an anti-nociceptive effect by directly hyperpolarizing the cell membrane and depressing pinch-evoked EPSCs in SG neurons.

### Analysis of postsynaptic action of Ultiva® in spinal cord slice preparations

**Fig 2:**
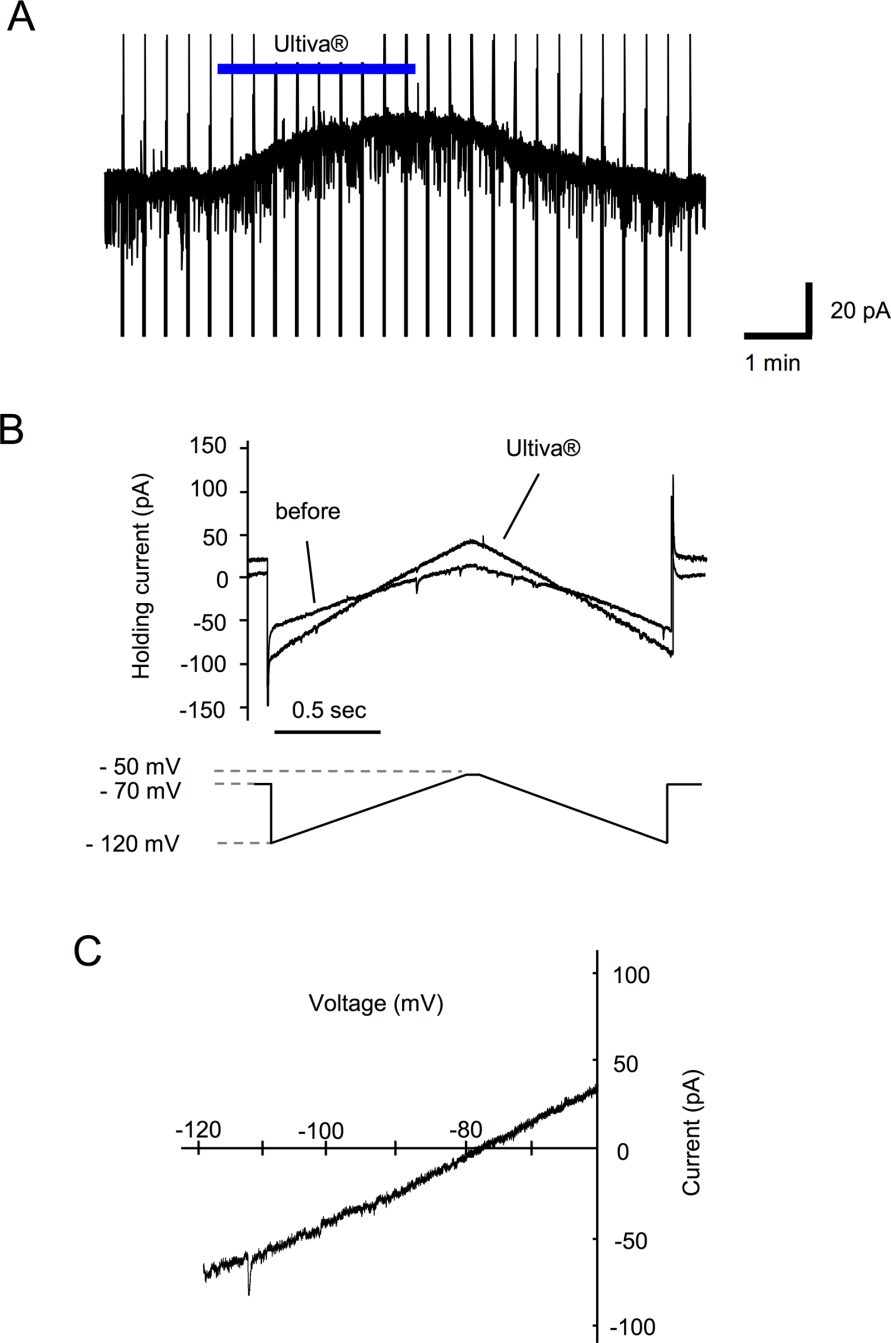
Voltage dependency of Ultiva® induced current. (A) Representative trace of Ultiva®-induced outward current in voltage-clamp mode (V_H_ = −50 mV) in spinal cord slice preparations. Vertical lines are traces of current response to voltage ramp commands. (B) Current response to the voltage ramp is shown over an expanded time base. The current-voltage relationship was recorded at the peak response to Ultiva® application. (C) Representative current-voltage relationship is plotted. The current-voltage relationship identified that the reversal potential of Ultiva® was −77.2 ± 1.6 mV (n = 27).

**Fig 3:**
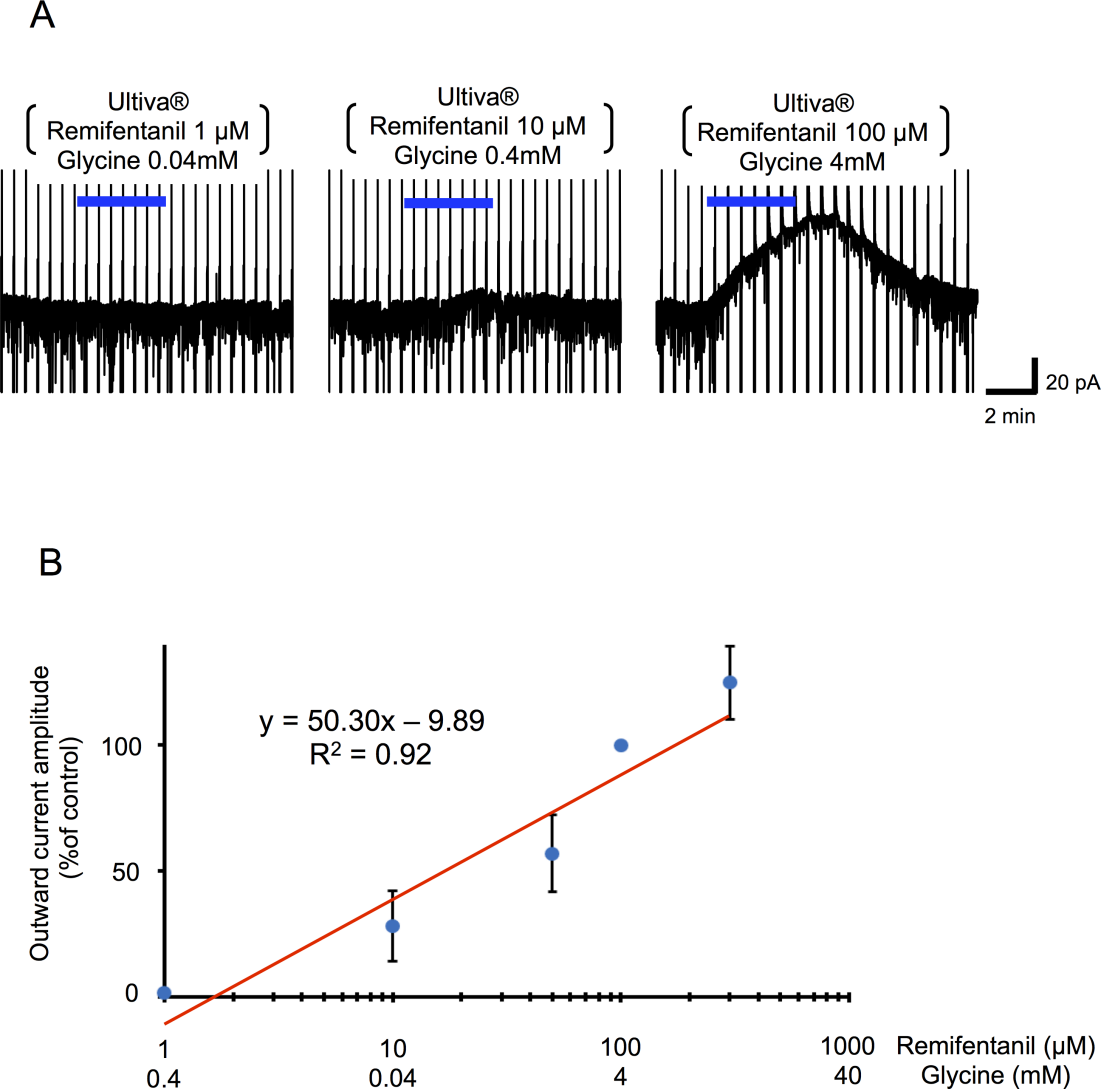
Dose-dependency of the Ultiva®-induced outward current. (A) Outward currents induced by Ultiva® at remifentanil concentrations of 1 μM, 10 μM and 100 μM. The readings shown were obtained from the same neuron. (B) Correlation between normalized amplitudes of Ultiva®-induced outward currents relative to those at 100 μM remifentanil and different concentrations of remifentanil and glycine (log scale). Each point with vertical bars represents the mean value and the standard error of the mean (n = 3–9). The coefficient, R^2^, was calculated as a measure of the relation between both variables (R^2^ = 0.92, p<0.01).

**Fig 4:**
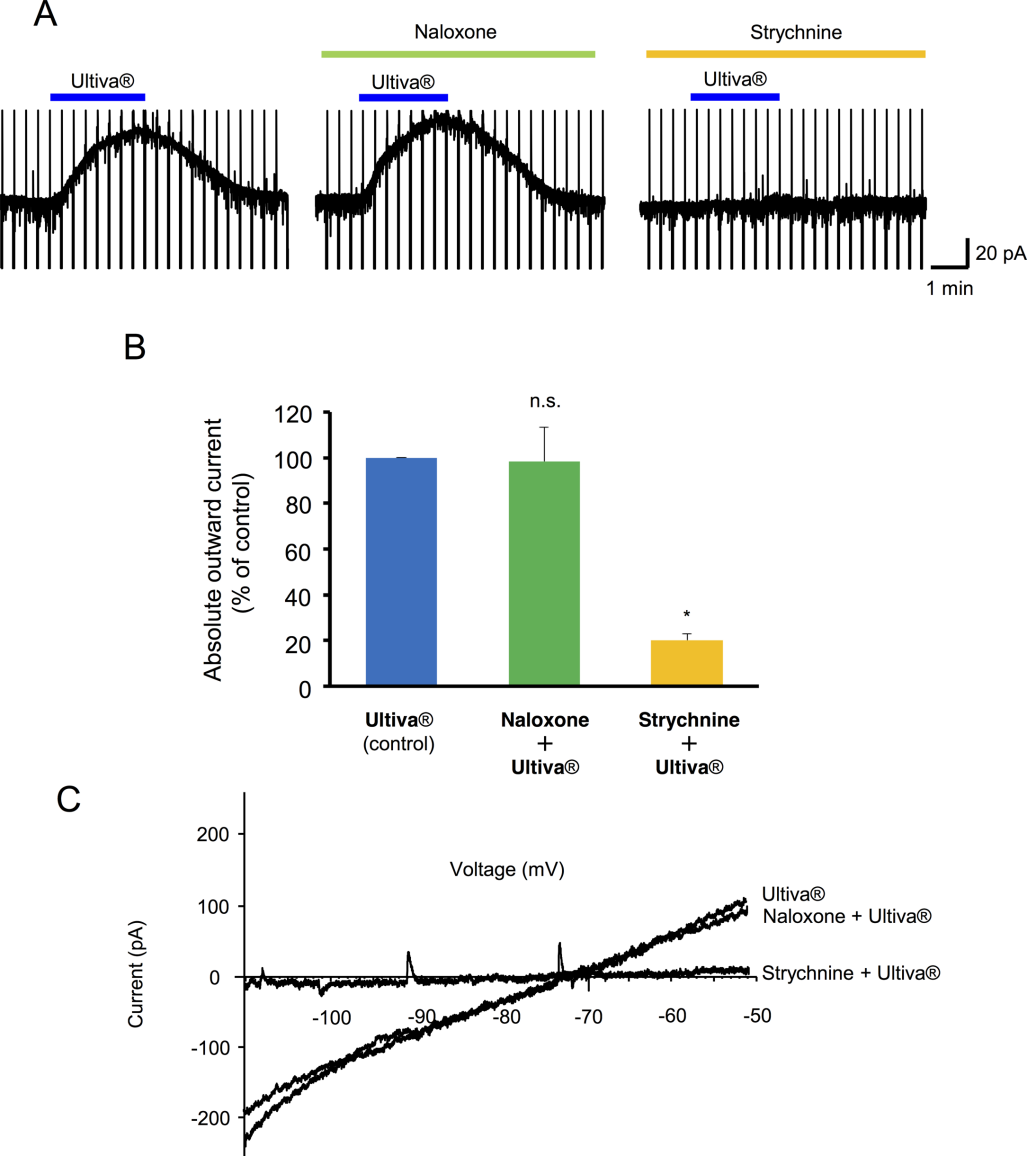
Effect of naloxone and strychnine on the postsynaptic actions of Ultiva®. (A) Representative current traces produced by Ultiva® in the presence of naloxone or strychnine. The Ultiva®-induced current was not depressed by naloxone (100 μM) but was abolished by strychnine (10 μM). The readings shown were obtained from the same neuron (n = 6, V_H_ = −50 mV). (B) Summary of normalized amplitudes of Ultiva®-induced outward currents are shown (Each bar, n = 6, n.s.; not significant). (C) Representative current-voltage relationship is plotted. The reversal potential of Ultiva® did not change in the presence of naloxone or strychnine.

**Fig 5:**
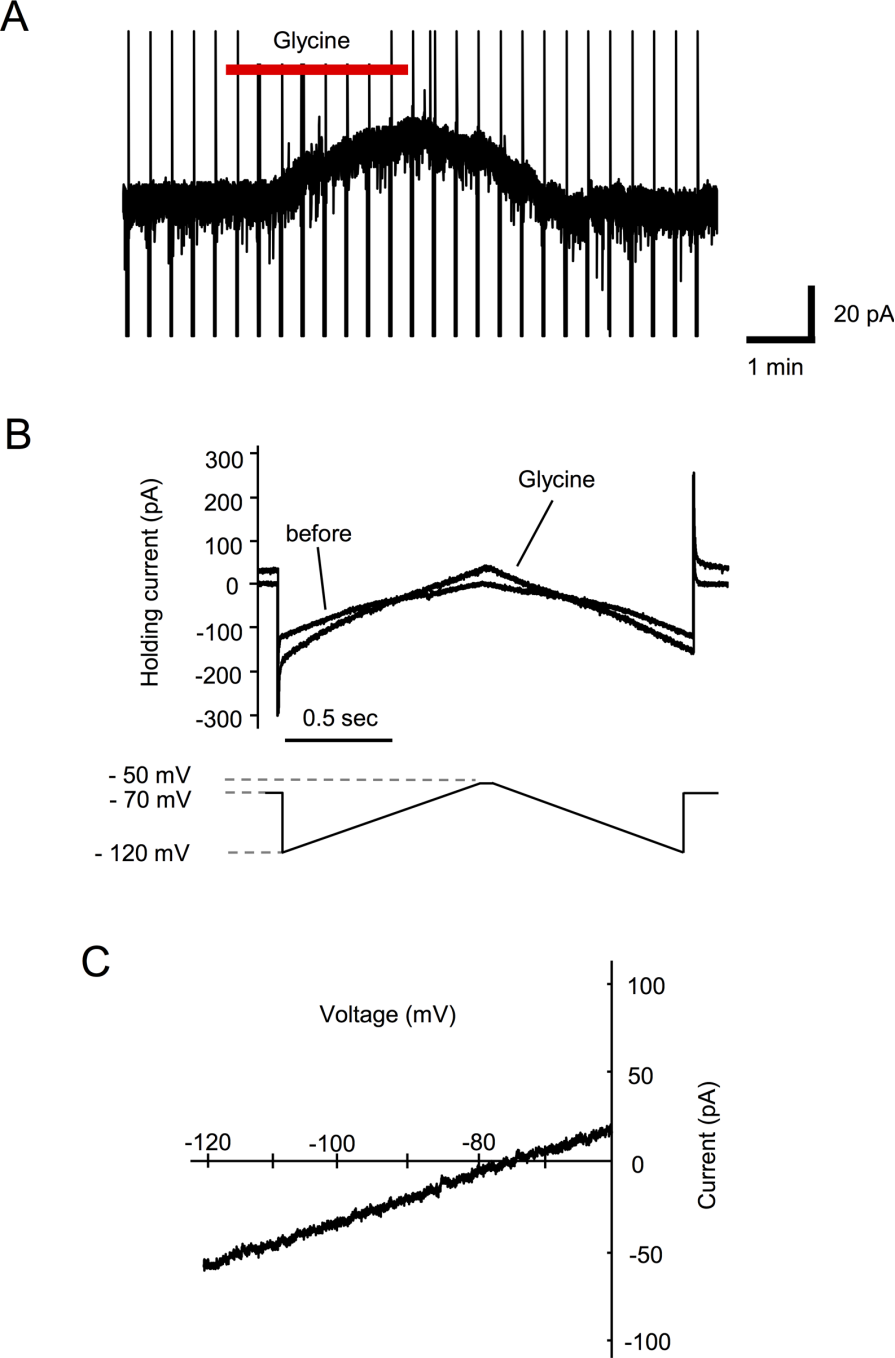
Voltage dependency of Glycine-induced current. (A) Representative trace of Glycine-induced outward current in voltage-clamp mode (V_H_ = −50 mV) in spinal cord slice preparations. (B) Current response to the voltage ramp is shown over an expanded time base. (C) Representative current-voltage relationship is plotted. The current-voltage relationship identified that the reversal potential of Glycine was −76.0 ± 2.5 mV (n = 9).

Perfusion of Ultiva® induced an outward current at a holding potential of −70 mV or −50 mV in spinal cord slice preparations, which was similar to the results obtained in the *in vivo* preparations (Fig 2A). An outward current was observed in 27 of 37 neurons (72.9%). The probability of response was different from the *in vivo* preparations. To examine the current-voltage relationships of the Ultiva®-induced current, triangle voltage ramp commands from −50 mV to −120 mV and back to −50 mV were applied before, during and after Ultiva® application every 20 s. The voltage ramp commands generated Ultiva®-induced current responses. The voltage sensitivity of the Ultiva®-induced current generated by the ascending ramp command was similar to that generated by the descending command (Fig 2B). After subtraction of the control current, the resulting ramp currents were plotted as a function of the command voltage (Fig 2C). The current-voltage relationships of these Ultiva®-induced currents had a mean reversal potential of −77.2 ± 1.6 mV (n = 27). There was also a dose-response relationship between the concentration of Ultiva® and the peak amplitude of Ultiva®-induced outward currents. The onset of responses was more rapid and the recovery was delayed as the concentration of Ultiva® increased (Fig 3A and 3B). Unexpectedly, the Ultiva®-induced outward current was not suppressed by μ-opioid receptor antagonist, naloxone (100 μM) in any of the neurons tested (n = 6; Fig 4A). In contrast, glycine receptor antagonist, strychnine (10 μM) suppressed the Ultiva®-induced outward current to 20.1 ± 2.8% of the control at a holding potential of −50 mV in the same neurons (P <0.001; Fig 4B). The reversal potentials of the Ultiva®-induced outward current did not change in the presence of naloxone (Fig 4C). The reversal potential of the Ultiva®-induced outward current was, however, almost the same as that of the current induced by 2 mM glycine (−76.0 ± 2.5 mV, n = 9, Fig 5A, 5B and 5C), which was close to the equilibrium potential of chloride (−73.2 mV) calculated from the Nernst equation using the concentrations of chloride in the internal and external solutions. The glycine-induced currents were suppressed by strychnine (10 μM) in all SG neurons tested. Glycine receptor is known to be ionotropic receptor that opens chloride channel and hyperpolarizes neurons. These findings suggest that the glycine included in the formulation of Ultiva® hyperpolarizes the postsynaptic membrane potential in SG neurons by activating chloride channels through glycine receptors.

### Analysis of presynaptic action of Ultiva® in spinal cord slice preparations

**Fig 6:**
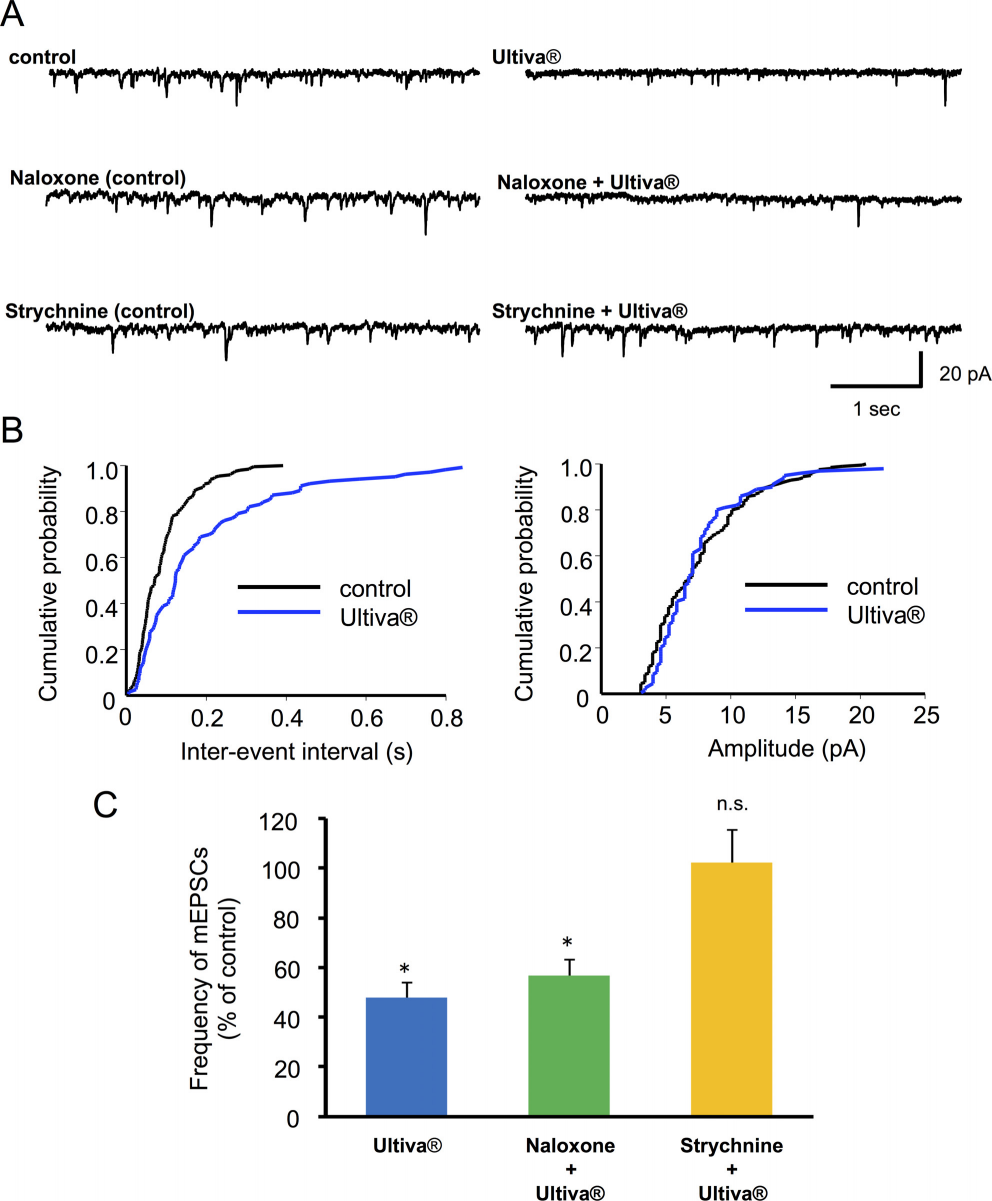
Effect of naloxone and strychnine on the presynaptic actions of Ultiva®. (A) Typical traces of miniature excitatory postsynaptic currents (mEPSCs). Ultiva® suppressed the frequency of mEPSCs (upper traces). Suppression of mEPSCs was observed in the presence of naloxone (100 μM, middle traces) but not in the presence of strychnine (10 μM, lower traces). The readings shown were obtained from same neuron (n = 6). (B) Cumulative distribution of the inter-event interval and mEPSC amplitude recorded before administration (black line) and in the presence of Ultiva® (blue line). Ultiva® did not affect the distribution of the amplitude (P = 0.27, right), but shifted the distribution to a longer inter-event interval (*P <0.001, left). (C) Summary of normalized frequencies of mEPSCs for each condition are shown (Ultiva®, n = 16; Ultiva® in the presence of naloxone, n = 13; Ultiva® in the presence of strychnine, n = 8).

**Fig 7:**
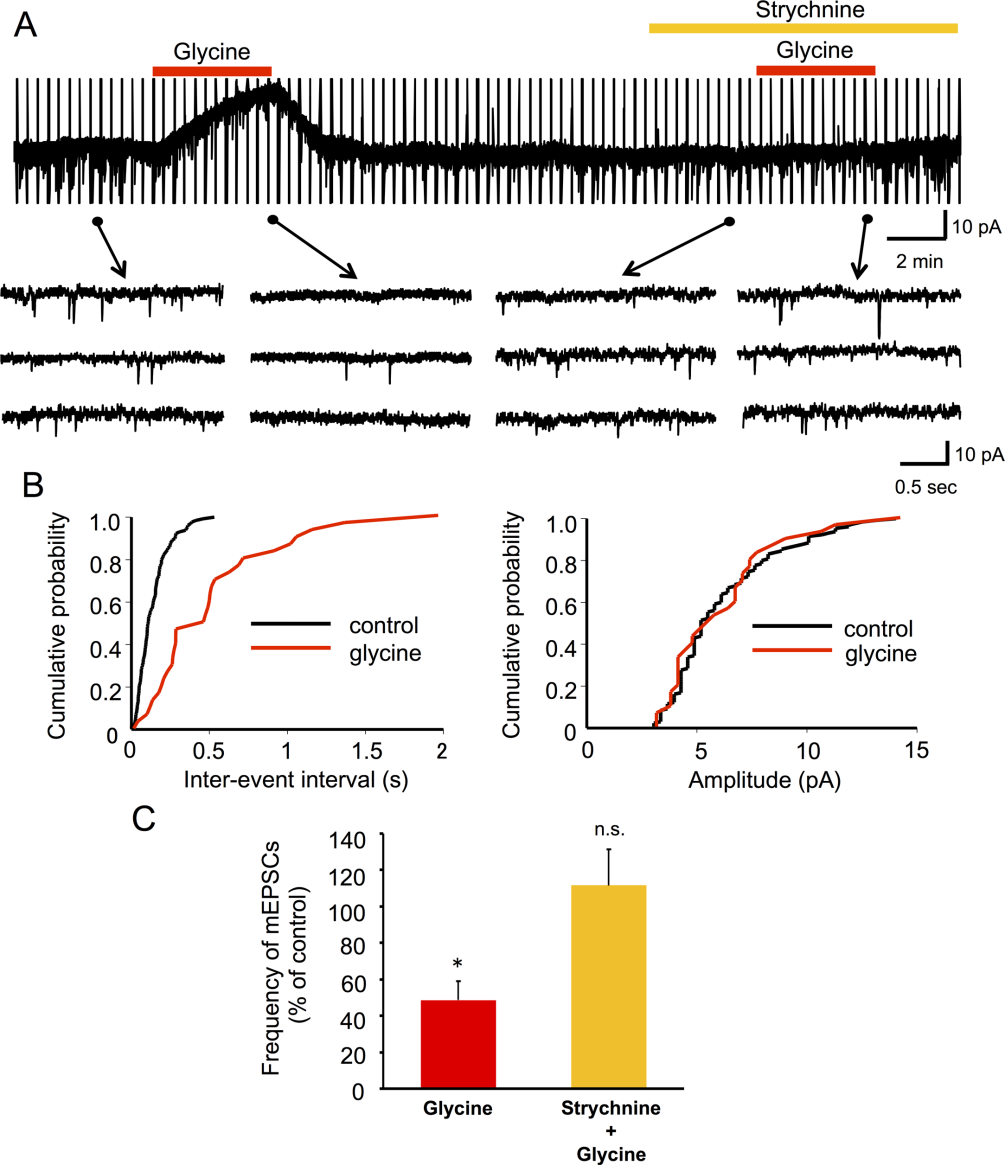
Effect of glycine on mEPSCs. (A) Representative traces of mEPSCs observed by glycine before (control), during administration, and in the presence of strychnine. Glycine (2 mM) suppressed the frequency of mEPSCs, but not in the presence of strychnine (10 μM). (B) Cumulative distribution of the inter-event interval and mEPSC amplitude recorded before administration (black line) and in the presence of glycine (red line). Glycine did not significantly affect the distribution of the amplitude (P = 0.30, right), but shifted the distribution to a longer inter-event interval (*P <0.001, left). (C) Summary of normalized frequencies of mEPSCs for each condition are shown (glycine, n = 11; glycine in the presence of strychnine, n = 5).

To investigate whether Ultiva® modulates glutamate release from presynaptic terminals, the effects of Ultiva® were examined on mEPSCs in SG neurons. The frequency of mEPSCs was significantly decreased by the application of Ultiva® (46.8 ± 6.6%, n = 16, P <0.001; Fig 6A, 6B and 6C) without affecting the amplitude (Fig 6B). Unexpectedly, naloxone (10–500 μM) did not significantly abolish the effect of Ultiva® on the frequency of mEPSCs in any of the neurons tested (56.8 ± 6.2% of control, n = 13, P <0.001; Fig 6A and 6C). Strychnine (10 μM), however, abolished the effect of Ultiva® on the frequency of mEPSCs in all neurons tested (102.2 ± 13.0% of control, n = 8; Fig 6A and 6C). Glycine (2 mM) inhibited mEPSCs (48.6 ± 10.3% of control, n = 11, P <0.001) in frequency but not amplitude (Fig 7A and 7B), an effect that was abolished by strychnine (10 μM) in all neurons tested (111.6 ± 19.8% of control, n = 5; Fig 7A and 7C). This suggests that the glycine contained in Ultiva® decreases glutamate release, and that the presynaptic action of Ultiva® is mediated by glycine receptors rather than opioid receptors.

## Discussion

We used *in vivo* preparations to show that the direct administration of Ultiva® to the surface of the spinal cord hyperpolarized SG neurons and suppressed the responses elicited by noxious stimuli applied to the receptive fields, together with spontaneous synaptic responses. These results confirm, as expected, that Ultiva® has an anti-nociceptive effect. Its mechanisms were further analysed *ex vivo* in spinal cord slices. Here, the glycine receptor antagonist strychnine suppressed Ultiva®-induced outward currents in SG neurons, but naloxone did not. In addition, Ultiva® decreased presynaptic glutamate release by the activation of glycine receptors, but not μ-opioid receptors. From these results, it would appear that intrathecal Ultiva® plays a major role in modulating nociception in the spinal cord by activating postsynaptic and presynaptic glycine receptors. Glycine is degraded by the glycine cleavage system in the liver after systemic administration, and the cerebrospinal fluid concentration of glycine is substantially lower than the plasma concentration in adult humans [17]. Glycine concentrations were found to be 9.5 μM in cerebrospinal fluid from clinical studies about intravenous Ultiva® in critical care patients [18], which is lower than the effective concentration of our results as shown in Fig 3. The glycine in intravenously administered Ultiva® might therefore be expected to be less influential on pain transmission in the central nervous system and spinal cord.

Behavioural studies with rats have demonstrated that intrathecal remifentanil has an analgesic effect, as does intrathecal glycine alone [1, 2]. These studies are partially consistent with our findings. Taken together with previous reports, our findings suggest that remifentanil might instead act at μ-opioid receptors in supraspinal structures. Consistent with this hypothesis, an effective intrathecal dose of remifentanil is reportedly accompanied by supraspinal side effects characteristic of opioids, such as impairment of the corneal and pinna reflexes [1, 2].

Agonists at the μ-opioid receptor inhibit synaptic transmission through both pre- and postsynaptic mechanisms in the spinal dorsal horn [19–21]. Our results raise the possibility that remifentanil, presented as Ultiva®, has a low affinity for μ-opioid receptors in the dorsal horn. We also demonstrated that exogenous glycine provoked outward currents and decreased presynaptic glutamate release in slice preparations. The SG is reported to contain glycine receptor-like immunoreactive neurons [22], and glycine generates outward currents by activating postsynaptic glycine receptors [23]. Although a morphological study implied that glycine receptors are located in primary afferent neurons in the cat dorsal horn [24], the pharmacologic or physiologic function of presynaptic glycine receptors in the SG is not well understood. In our study, both Ultiva® and exogenous glycine decreased the frequency of mEPSCs, an effect that was abolished by strychnine. Thus, we presume that exogenous glycine is involved in presynaptic inhibitory processes. Further studies are required to elucidate this presynaptic action of glycine in SG neurons.

Some investigators have highlighted the risk that Ultiva® may provoke OIH, described as increasing pain sensitivity involving sensitization of pro-nociceptive pathways [25]. Human and animal studies report that intraoperative Ultiva® administration can provoke hyperalgesia in the postoperative period and may increase analgesic requirements [6, 26–31], but there are also reports to the contrary [32–34]. Although it is still to be established whether Ultiva® induces hyperalgesia, it is proposed that it might do so by activating NMDA receptors. Ultiva® directly activates human NMDA receptors expressed in *Xenopus laevis* oocytes [16], and also indirectly enhances NMDA receptor function in cultured rat embryonic dorsal horn neurons [6]. A study using rat spinal cord slice preparations also reported that the glycine contained in Ultiva® activated NMDA receptors on dorsal horn neurons [5]. We found, however, that Ultiva® had no excitatory effects on SG neurons during administration to the spinal cord. These discrepancies might be explained by our choice of *ex vivo* specimen, or route, dose or duration of administration. Vanderah *et al*. have reported that the rostral ventromedial medulla in the brainstem mediates OIH [35, 36]. Intravenous administration of Ultiva® might therefore activate supraspinal structures, or might indirectly affect NMDA receptors in the spinal cord.

Although we have shown that glycine contained in Ultiva® acts as anti-nociceptive agent, glycine could affect motor neurons in the event of accidental spinal administration. Accidental epidural administration of Ultiva® reportedly impairs conscious level and respiratory function, and causes muscle rigidity [37]. Intrathecal glycine brings about reversible motor impairment in a rat behavioural model. Although there would likely be substantial pharmacological and pharmaceutical challenges, the glycine receptor in the spinal cord could represent a new target for drug development.

In conclusion, Ultiva® hyperpolarizes spinal SG neurons by activating chloride channels through glycine receptors, but it appears that remifentanil had little effect on μ-opioid receptors. Our findings suggest that intrathecal administration of Ultiva® may mediate its anti-nociceptive effects by a variety of means in the spinal cord as well as in supraspinal structures, and its anti-nociceptive mechanisms may be different from that of intravenous administration.
